# Malignancy in Systemic Sclerosis: A Multicenter Retrospective Study

**DOI:** 10.3390/biomedicines13040993

**Published:** 2025-04-19

**Authors:** Dóra Nemes-Tömöri, Dávid Kurszán Jász, Dóra Tari, Bernadett Bói, Ágnes Ágoston-Szabó, Gabriella Szűcs, Gyöngyike Emese Majai

**Affiliations:** 1 Division of Clinical Immunology, Institute of Internal Medicine, Faculty of Medicine, University of Debrecen, Móricz Zsigmond Road 22, 4032 Debrecen, Hungary; mgyongyi@med.unideb.hu; 2 Department of Rheumatology and Immunology, Medical School, University of Pécs, Akác Street 1, 7632 Pécs, Hungary; jasz.david@pte.hu (D.K.J.); szabo.agnes@pte.hu (Á.Á.-S.); 3 Department of Rheumatology and Immunology, Faculty of Medicine, University of Debrecen, Móricz Zsigmond Road 22, 4032 Debrecen, Hungary; tari.dora@med.unideb.hu (D.T.); szucs.gabriella@med.unideb.hu (G.S.); 4 Department of Public Health and Epidemiology, Faculty of Medicine, University of Debrecen, Kassai Road 26, 4028 Debrecen, Hungary; boi.bernadett@med.unideb.hu

**Keywords:** systemic sclerosis, scleroderma, cancer, malignancy, skin cancer

## Abstract

**Background/Objectives**: Systemic sclerosis (SSc) is associated with high malignancy risk. With improving SSc management, tumor risk could change, therefore re-evaluating the possibility of neoplasms is necessary. Our aim was to observe malignancy prevalence and its risk factors in the Hungarian SSc population, comparing them to our previous and international results. **Methods**: We retrospectively collected the data of SSc patients followed by and admitted to three Hungarian clinical centers between 2018 and 2024. The collected data included the characteristics of SSc and neoplasms, autoantibody positivities, immunosuppressive treatments, pregnancy and environmental factors. **Results**: Out of 541 patients, 85 had malignancy and, in total, 96 tumors were registered. Skin cancer was the most common (*n* = 24), followed by breast (*n* = 14) and lung cancer (*n* = 14). Among skin cancers, almost one-third was melanoma. Tumors mostly appeared in two peaks: around the time of SSc diagnosis and 10 years later. The occurrence of anti-RNA Polymerase III (anti-RNAPIII) was significantly higher in cancerous patients. Tumor risk was higher with anti-RNAPIII (Odds Ratio (OR) 4.33, 95% Confidence Interval (95% CI) 1.08, 15.1) and anti-topoisomerase I (ATA) (OR 2.34, 95% CI 0.94, 5.84) positivity. Women and patients with diffuse cutaneous SSc (dcSSc) were more likely to have malignancy. Smoking (OR 1.27, 95% CI 0.53, 3.00) also raised the possibility of carcinogenesis. Cancerous patients were older (*p*-value = 0.003), and their mortality was worse compared to non-cancerous patients (Hazard Ratio (HR) 4.75, 95% CI 2.12, 10.62). Pregnancy did not provide a protective effect against breast cancer. **Conclusions**: Malignancy significantly contributes to the increased mortality in SSc. Female gender, dcSSc, anti-RNAPIII positivity, smoking and older age represent a higher risk of tumors. Dermatological cancer screening is necessary for all patients with SSc.

## 1. Introduction

Systemic sclerosis is a complex autoimmune disease whose pathogenesis revolves around the triad of fibrosis, vasculopathy and autoimmunity, ultimately leading to multi-organ complications [1,2].

While it can affect individuals of any age, it most commonly develops in middle-aged adults, with studies reporting variations in the peak age of onset [3,4,5]. Notably, the condition occurs more frequently in women than in men.

The pathogenesis of SSc involves a combination of triggers, including exposure to environmental agents such as silica, organic solvents, and vinyl chloride [6,7,8,9,10,11,12]. These factors, alongside genetic predisposition, contribute to the development and progression of the disease [1].

In addition to its direct effects, systemic sclerosis is associated with an increased risk of malignancies. This risk varies depending on the disease subtype, with patients suffering from dcSSc being particularly vulnerable to developing tumors [13].

Research has shown an overall higher risk for lung, breast, gastrointestinal, skin cancer and hemopoietic malignancies [14,15,16], which (partly) is in accordance with previously published Hungarian data [17,18]. The increased incidence of malignancies in SSc has a multifactorial origin. The pathogenesis of systemic sclerosis may promote the formation of malignant tumors. Common steps in pathogenesis may be responsible for the connection between lung cancer, breast cancer, melanoma and SSc.

Therapeutic approaches for systemic sclerosis focus on its clinical manifestations and encompass immunosuppressive, antifibrotic and vasoactive agents [19]. However, evidence suggests that immunosuppressive therapy may contribute to a higher malignancy risk in SSc patients [16,20].

The etiology of malignancy in systemic sclerosis involves a complex interplay of risk and protective factors. Epidemiological studies have identified specific environmental carcinogens, including vinyl chloride and organic solvent exposure, as significant risk factors for oncogenesis [21,22,23,24,25]. In contrast, emerging evidence suggests that parity may exert a protective effect against breast cancer development, potentially mediated through endocrine and immunological mechanisms [26]. These observations underscore the critical need for (1) systematic screening for environmental exposures in high-risk cohorts, and (2) further mechanistic studies to elucidate the putative protective relationship between pregnancy and breast cancer pathogenesis in systemic sclerosis.

The mortality among SSc patients is higher than the general population [27]. Considering the mortality of Hungarian SSc patients, organ involvement and malignancies are important causes of death [18,28].

The aim of this study is to assess three Hungarian clinical centers’ database to determine the occurrence of tumors in SSc, risk or protective factors for carcinogenesis. Our goal was to obtain a comprehensive understanding of the malignancy risk in Hungarian patients with systemic sclerosis by not only gathering data on the characteristics of the underlying disease and neoplasms but also identifying risk factors that allow the recognition of higher tumor risk patients.

These findings carry significant clinical implications for malignancy screening protocols and early detection strategies in systemic sclerosis. Currently, there exists a notable gap in large-scale epidemiological data characterizing tumor risk patterns within the Hungarian population.

## 2. Materials and Methods

### 2.1. Study Population

In our retrospective study, we collected the data of SSc patients from the Division of Clinical Immunology, Institute of Internal Medicine, Department of Rheumatology and Immunology of the University of Debrecen and Department of Rheumatology and Immunology of the University of Pécs between 2018 and 2024.

Adult patients were included if they fulfilled the ACR (American College of Rheumatology) or 2013 ACR/EULAR (American College of Rheumatology; European League Against Rheumatism) classification criteria according to the date of diagnosis [29,30].

The study was conducted in accordance with the Declaration of Helsinki and approved by the Regional Research Ethics Committee of University of Debrecen (protocol number RKEB-6727-2024, 24 January 2024) and the Scientific and Research Ethics Committee of the Scientific Council for Heath (protocol number BM/8466-1/2024, 5 April 2024).

### 2.2. Laboratory Evaluation

Immunoserological parameters were determined from the serum samples. The presence of antinuclear antibodies (ANA) was detected using the immunofluorescence method. Immunoblot analysis was performed for detection of ATA, anti-centromere (ACA), anti-RNAPIII, anti-PM/Scl-75 and 100, anti-Ku, anti-fibrillarin, anti-Th/To, anti-NOR90 and anti-PDGF. All laboratory tests were performed at the Department of Laboratory Medicine, Faculty of Medicine, University of Debrecen and at the Department of Laboratory Medicine, Faculty of Medicine, University of Pécs.

### 2.3. Collected Data

- Demographic: date of birth, age, sex, presence and numbers of pregnancy.
- Clinical: year of SSc diagnosis, SSc subtype (limited or diffuse), year of death, cause of death, year of malignancy diagnosis, malignancy type and outcome of malignancy.
- Serological: immunoserological positivity with special emphasis on ANA, anti-RNAPIII, ACA, ATA, anti-PM/Scl-75 and 100, anti-Ku, anti-fibrillarin, anti-Th/To, anti-NOR90 and anti-PDGF.
- Treatment-related: immunosuppressive treatments, therapeutic indications and duration of treatments.
- Risk and environmental factors: smoking; alcohol consumption with the subcategories of ‘no’, ‘sometimes’, ‘occasionally’ and ‘regularly’; organic solvents; silica; vinyl chloride exposures and immunosuppressive treatments.

### 2.4. Statistical Analysis

Values are expressed as mean and standard deviation (SD) or median with interquartile range (IQR) for continuous variables, and frequency with percentage for categorical variables. Kolmogorov-Smirnov and Shapiro–Wilk normality tests were used to determine the distribution of data. Continuous variables were compared with parametric two-sample *t*-test or nonparametric Mann–Whitney U test for two samples. Categorical variables were compared with Pearson’s chi-squared test or Fisher’s exact test. Pearson correlation was used to test the association between each data series. Univariate and multivariate logistic regression analysis was performed to identify protective or risk factors for tumor occurrence, listed odds ratio for the covariates. Kaplan–Meier method was used to analyze survival, Log-rank test was used to compare the survival curves and hazard ratio was calculated to compare the hazard between the groups. All statistical tests were two-sided and differences were considered statistically significant at <0.05 level and reported using *p*-values and/or 95% confidence intervals. Statistical analysis was performed with R programming language (RStudio: Integrated Development for R version 2024.09.0, RStudio PBC, Boston, MA, USA) using the *gtsummary* package [31]. Figures were generated using GraphPad Prism for Windows, version 10.3.1 (GraphPad Software, Boston, MA, USA).

## 3. Results

### 3.1. Demographic Characteristics

In this study, the data of 541 patients were evaluated. The most frequent type of systemic sclerosis was limited cutaneous form (lcSSc) with 62.5% of the cases (*n* = 338) and 37.5% were diffuse cutaneous form (*n* = 203). The distribution of the patients was as follows: cancerous: lcSSc *n* = 47 (55.3%), dcSSc *n* = 38 (44.7%), non-cancerous: lcSSc *n* = 291 (63.8%), dcSSc *n* = 165 (36.2%). The duration of SSc at the end of the study period was almost identical in our overall patient pool (13.6 ± 9.4 years) and in cancerous (13.9 ± 10.3 years) and non-cancerous (13.6 ± 9.2 years) patients.

Regarding genders, female predominance was observed, i.e., 85.6% of the patients were women (*n* = 463). The average age in this cohort was 63.2 ± 12.4 years. Comparing the average age of cancerous and non-cancerous patients, cancerous patients were older at the end of the study period (66.8 ± 11.9 years vs. 62.5 ± 12.4 years, *p* = 0.003) and also at the diagnosis of SSc compared to non-cancerous patients (52.6 ± 13.9 years vs. 48.5 ± 13.2 years, *p* = 0.009).

**Figure 1:**
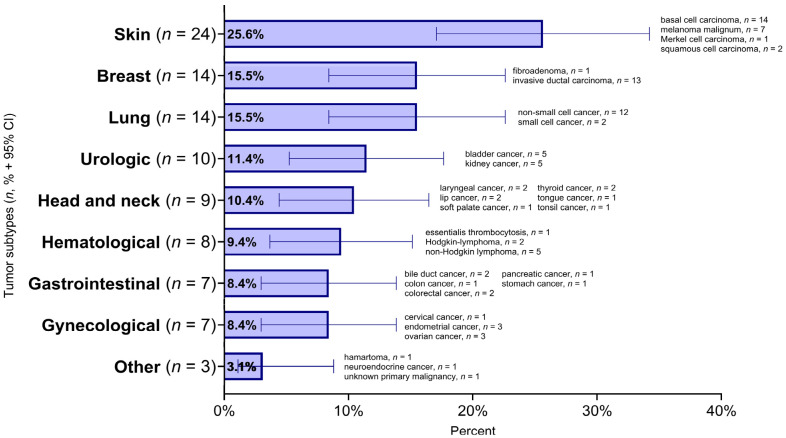
Prevalence of tumor types (*n* = 96) and histological subtypes among SSc patients (*n* = 85) 95% CI: 95% confidence interval.

In 85 cancerous patients (15.7% of patients), 96 tumors were diagnosed. Ten patients had two, and one patient had three malignancies in their medical history. Tumor development did not influence the distribution of genders; most of the patients with tumors were female (*n* = 74, 87.1%). All in all, 9 types of malignant diseases were detected in cancerous patients (skin, breast, lung, urologic, head and neck, hematological, gastrointestinal, gynecological and other). Skin cancers were significantly more common than all the other tumor types (*n* = 24, 25.6%), except for breast, lung and urologic cancers (Figure 1). This was followed by the malignancies of breast (*n* = 14, 15.5%) and lung (*n* = 14, 15.5%). Urologic (*n* = 10, 11.4%), head and neck (*n* = 9, 10.4%) and hematological malignancies (*n* = 8, 9.4%) appeared in similar proportions in tumor positive SSc patients. However, the number of gastrointestinal (*n* = 7, 8.4%) and gynecological (*n* = 7, 8.4%) tumors were the same. One hamartoma and one neuroendocrine cancer were registered. In the case of one patient, the primary malignancy remained unknown until recently. Regarding skin cancers, after basal cell carcinoma (*n* = 14), malignant melanoma was the second most prevalent cutaneous malignancy (*n* = 7). Skin and gynecological cancers were presented more frequently in patients with limited cutaneous systemic sclerosis.

### 3.2. Temporal Relationship of SSc and Malignancy

**Figure 2:**
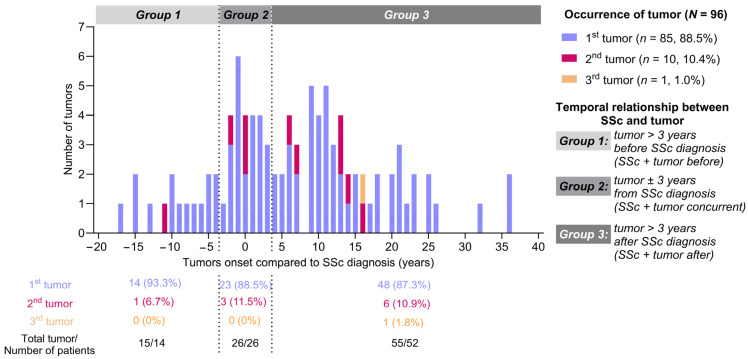
General characteristics of tumor events. Significant time differences can be observed between the diagnosis of malignancy and SSc. Calculation of elapsed time: the difference between the year of malignancy and SSc diagnosis. SSc: systemic sclerosis.

Considering the possibility of malignancy-induced autoimmunity, the onset of tumors was examined in 3-year periods; therefore, three groups were defined: tumor > 3 years before SSc diagnosis (Group 1), tumor ± 3 years from SSc diagnosis (Group 2) and tumor > 3 after SSc diagnosis (Group 3) (Figure 2).

**Figure 3:**
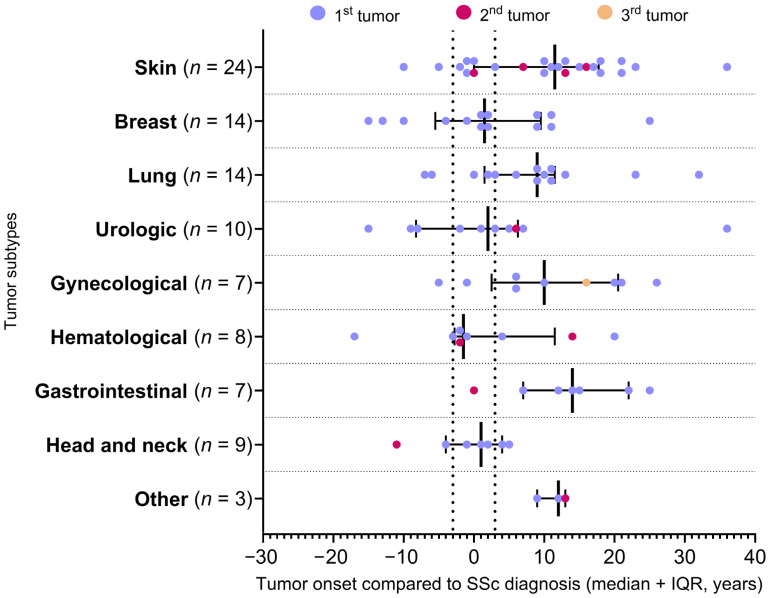
Temporal relationship between SSc and tumor types. Calculation of the elapsed year(s): the difference between the year of the tumor and the diagnosis of SSc. Dotted lines represent 3 years before and after SSc diagnosis. SSc: systemic sclerosis, IQR: interquartile range.

The greatest incidence of tumors was found in Group 3 (48/85 tumors, 56.5%), while there were half as many cases in Group 2 (23/85 tumors, 27.1%). The smallest proportion were tumor cases in Group 1 (14/85, 16.5%). The second malignancies were also more frequent in Group 3 (6/10, 60.0%), followed by Group 2 (3/10, 30.0%). The third tumor was diagnosed in Group 3. Considering the time course of the onset of tumors, two peaks can be observed. The first peak was around the diagnosis of systemic sclerosis, covering Group 2 and the second peak was 10 years after the diagnosis of SSc (Figure 3).

### 3.3. Autoantibodies and Malignancy

Antinuclear antibodies, including ATA, anti-Ro52 and ACA were the most common in the entire population studied. Anti-RNAPIII positivity occurred more than twice as often among patients with tumor (12.9% vs. 5.5%, *p* = 0.012). The majority of non-cancerous patients were more often negative for the autoantibodies (15.7% vs. 3.5%, *p* = 0.003). There was no anti-PDGF positivity among cancerous patients.

**Figure 4:**
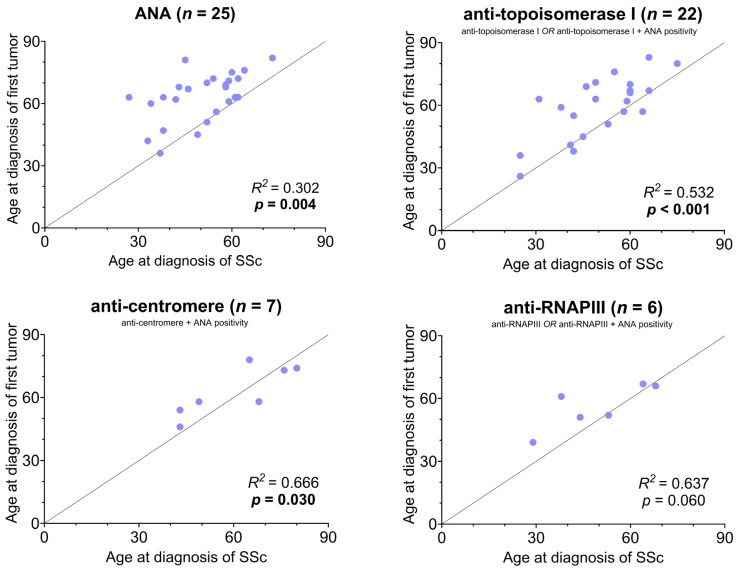
Relationship between age at first tumor diagnosis and SSc onset according to antibody positivity. The dotted line indicates the complete coincidence of the age at diagnosis of the first tumor and SSc. The relationship between the age at the time of diagnosis of the first tumor and the age at the time of diagnosis of SSc was represented according to the individual antibody positivities, in which the number of cases was at least 5 (on its own or with ANA positivity). SSc: systemic sclerosis, ANA: antinuclear antibodies, anti-RNAPIII: anti-RNA Polymerase III.

Regarding diffuse and limited cutaneous scleroderma cancerous patients, ATA occurred more frequently in diffuse cutaneous tumor positive patients (50% vs. 14.9%, *p* < 0.001), while ACA happened to be more prevalent in cancerous patients with lcSSc (23.4% vs. 2.6%, *p* = 0.006). Similar distribution was observed in non-cancerous patients. We examined the four most common immunoserological positivities depending on the age at diagnosis of SSc and malignancy. In Figure 4, the relationship between the age at the diagnosis of the first tumor and the age at the onset of SSc was depicted according to the individual antibody positivities. The Pearson correlation was significant for antinuclear antibodies, and for two of them, ACA and ATA. In the case of anti-RNAPIII, the correlation was strong, but not conclusive.

Notably, almost half of all anti-PM/Scl-75 and 100 autoantibody positivities (*n* = 3, 12.5%) were registered in skin cancer. Melanoma and anti-PM/Scl were observed together only once (Table S1).

### 3.4. Treatment Modalities and Malignancy

**Figure 5:**
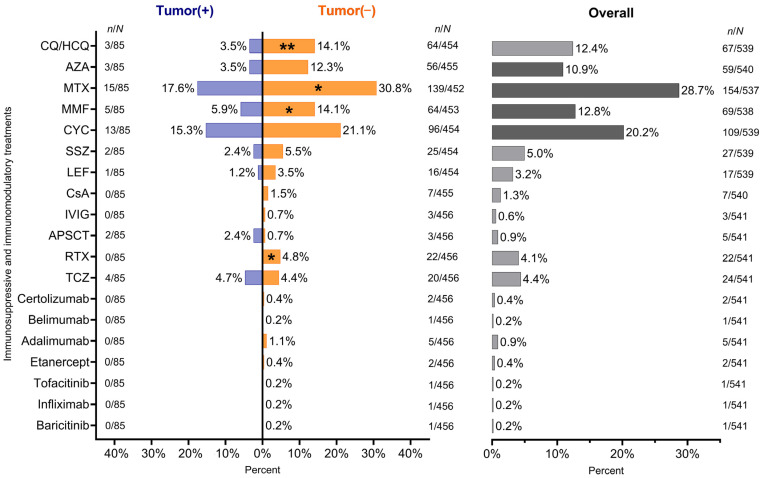
Immunosuppressive and immunomodulatory treatment in the SSc cohort and their comparison between cancerous and non-cancerous patients. In the case of cancerous patients, treatments before the diagnosis of the tumor were considered. Significant *p*-values comparing patients with malignancy versus without malignancy are shown as follows: *: *p* < 0.05; **: *p* < 0.01. CQ: chloroquine, HCQ: hydroxychloroquine, AZA: azathioprine, MTX: methotrexate, MMF: mycophenolate mofetil, CYC: cyclophosphamide, SSZ: sulfasalazine, LEF: leflunomide, CsA: cyclosporine A, IVIG: intravenous immunoglobulin, APSCT: autologous peripheral stem-cell transplantation, RTX: rituximab, TCZ: tocilizumab.

Methotrexate (28.7%), cyclophosphamide (20.2%), mycophenolate mofetil (12.8%) and azathioprine (10.9%) were the most frequently given in our patients (Figure 5). The same treatment distribution was observed regarding tumor negative patients. Twenty-seven patients were treated with at least one immunosuppressive drug prior to tumor onset (Table S2). Among non-tumor patients, chloroquine/hydroxychloroquine (14.1% vs. 3.5%, *p* < 0.01), methotrexate (30.8% vs. 17.6%, *p* < 0.05), mycophenolate mofetil (14.1% vs. 5.9%, *p* < 0.05) and rituximab (4.8% vs. 0%, *p* < 0.05) were administered more often compared to cancerous patients.

**Figure 6:**
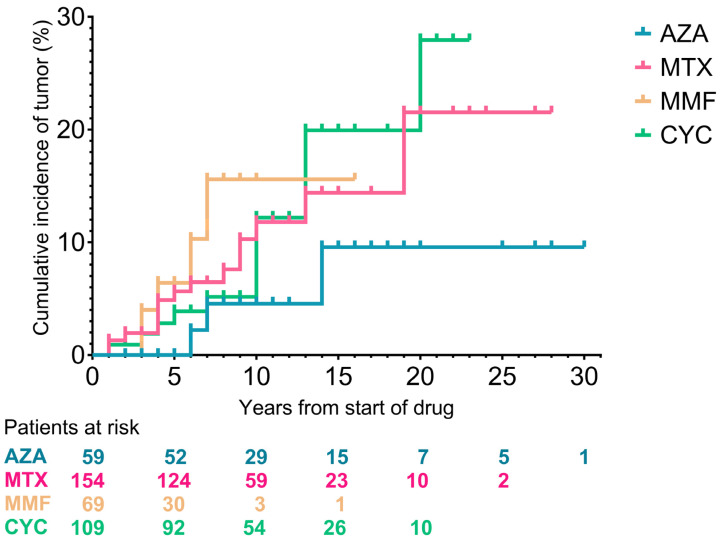
Cumulative incidence of malignancies depicted by immunosuppressive drugs. AZA: azathioprine, MTX: methotrexate, MMF: mycophenolate mofetil, CYC: cyclophosphamide.

Evaluating the correlation between tumor occurrence and immunosuppressive drugs in a 20-year period, the cumulative incidence of tumors was the highest after the administration of cyclophosphamide and methotrexate. Cumulative incidence was relatively lower in patients receiving mycophenolate mofetil and azathioprine, but all these results were not significant (Figure 6).

### 3.5. Environmental Factors and Malignancy

In this study, we aimed to investigate several exposures in relation to malignancy development. In Table 1, the numbers show that, in numerous cases, information about the different exposures was missing in each patient’s medical history. Using the available data, it can be stated that the frequency of smoking was lower in non-cancerous patients (39.9% vs. 42.1%, *p* = 0.752). However, alcohol consumption was more prevalent in this group (5.6% vs. 10.7%, *p* < 0.001). No significant difference was found between cancerous and non-cancerous patients regarding organic solvents. No patient had a history of exposure to silica or vinyl chloride. In terms of gender, smoking (63.9% vs. 35.3%, *p* < 0.001) and alcohol consumption (16.1% vs. 8.5%, *p* = 0.297) were more common in men. Pregnancy was not associated with a reduced incidence of breast cancer in our study, indeed all the patients with breast cancer were pregnant. In 9 cases of the 14 patients with breast cancer, data about gynecological history were available.

### 3.6. Risk Factors for Malignancy

**Figure 7:**
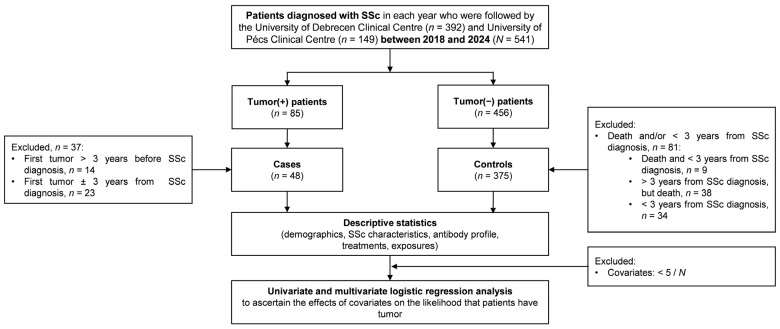
Flow chart for the examination of risk factors for tumor. SSc: systemic sclerosis.

Logistic regression was used to investigate the risk factors of malignancy; its structure can be seen in Figure 7.

Based on the results of the univariate logistic regression analysis (Model 1), we have observed a higher chance of tumor formation in case of anti-RNAPIII positivity (OR 3.18, 95% CI 1.09, 8.20). Female sex (OR 1.20, 95% CI 0.52, 3.27) and dcSSc (OR 1.44, 95% CI 0.78, 2.63) also increased the risk of malignancies. No relationship was perceived between the tumor and the age at diagnosis of SSc and the duration of SSc. A higher probability of carcinogenesis was found in the case of ANA (OR 2.17, 95% CI 0.96, 5.84), ATA (OR 1.36, 95% CI 0.69, 2.58), as well as smoking (OR 1.05, 95% CI 0.47, 2.27), while it was lower for ACA and anti-Ro52. In this analysis, neither methotrexate nor cyclophosphamide did not increase the likelihood of tumors. Multivariate logistic regression analysis (Model 2) including autoantibodies, treatments and smoking brought similar results. After demographic and SSc characteristics adjustment (Model 3) results were like Model 2; however, cyclophosphamide seemed to be a risk factor for tumor development (OR 1.09, 95% CI 0.38, 2.83) (Table 2).

### 3.7. Survival of Patients with SSc and Malignancy

**Figure 8:**
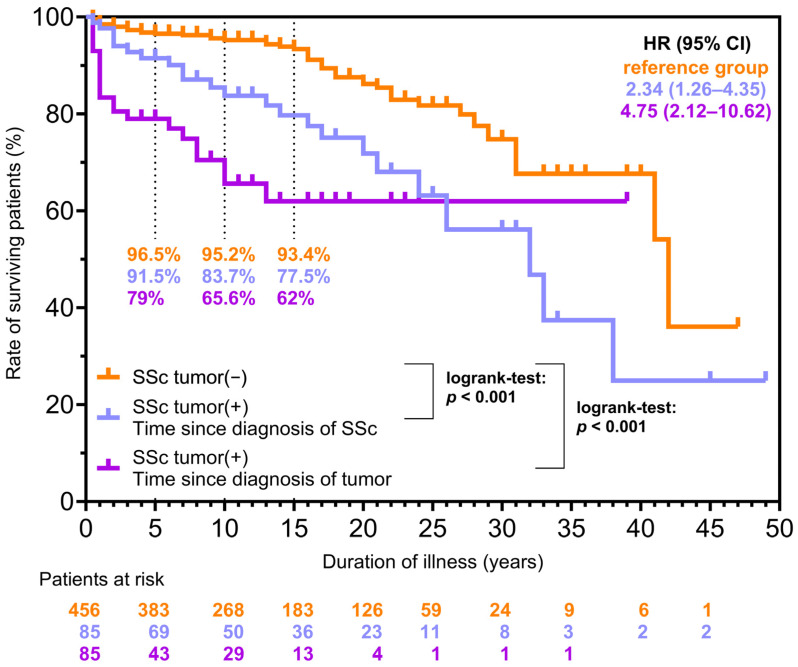
Kaplan–Meier survival curves depicting the estimated survival probability of cancerous and non-cancerous SSc patients. In the case of non-cancerous and cancerous patients, the follow-up time is the difference between death or the end of the study period and the diagnosis of SSc (SSc tumor(−) and SSc tumor(+) time since diagnosis of SSc). In cancerous patients, instead of the diagnosis of SSc, there is also an approach to the year of tumor diagnosis (SSc tumor(+) time since diagnosis of tumor). SSc: systemic sclerosis, HR: hazard ratio, 95% CI: 95% confidence interval.

Out of the 85 cancerous patients, 23 died, 11 due to malignancy and its complications. Tumor associated deaths occurred in the case of lung, breast, kidney, tonsil, bile duct, gastric and colon cancer. The overall mortality of cancerous SSc patients was considerably worse compared to non-cancerous patients (Figure 8). The rate of surviving patients dropped rapidly after the diagnosis of tumor. Using non-cancerous patients as a reference group, after the diagnosis of SSc, the risk of death for patients with malignancy was twice as high (HR: 2.34, 95% CI: 1.25–4.36, *p* < 0.001). It was four times higher after tumor diagnosis (HR: 4.75, 95% CI: 2.12–10.62, *p* < 0.001).

## 4. Discussion

It is known that the diagnosis of systemic sclerosis comes with an overall increased risk of malignancies, but there are differences between the publications regarding the type of tumors, which may be due to the heterogenity of the studied population [32,33,34,35].

Overall, the occurrence of breast and lung cancer is frequently reported in the literature, including in our most recent study. However, in our studied SSc population, skin cancer was the leading tumor type, melanoma being the second most common. In our work, a close temporal relationship to SSc and tumor onset was observed in two peaks, emphasizing the importance of screenings throughout the whole patient follow-up. Anti-RNAPIII positivity, especially in older patients with dcSSc, increases significantly the risk of malignancies. Patient survival after the diagnosis of SSc and tumor deteriorated rapidly.

A meta-analysis from Bonifazi et al. estimated an approximately 75% increased risk for developing malignancies compared to the average population and in details a meta-analysis of population based cohort studies reported an especially higher risk for lung, liver, hematological and bladder malignancies [14,32].

Interstitial lung disease, due to fibrosis, appeared to elevate the chance of lung cancer. Peng et al. reported significantly higher risk for lung cancer with OR 2.80 (95% CI 1.55, 5.03) [24]. Liver cancers in SSc were explained with the higher prevalence of primary biliary cholangitis. In other studies, breast cancer was the most observed cancer, mostly followed by lung and hematologic malignancies [33,34,36]. A French study revealed that the majority, approximately one-third of malignancies, among SSc patients were breast cancers followed by lung and gastrointestinal cancers [16].

Considering the Hungarian SSc population, in a previous study published by our co-authors the prevalence of all cancers were 4.6% and among these patients lymphoma, lung and breast cancer were the most frequent types of malignancies, respectively, from 218 SSc patients, three had non-Hodgkin lymphoma, two had lung cancer and two patients had breast malignancy [17]. In the general Hungarian population, the most common type of malignancies are the neoplasms of the skin and 13.1% of them are melanoma. The second most prevalent tumor type is urological cancers (bladder, kidney, urethral, ureteral, prostate and penile), followed by lung and breast cancer [37].

Previously reported studies showed that skin cancers are not as frequent; however, Morrisroe et al. reported melanoma as the second most predominant tumor type in SSc patients [33].

In our study, in the case of skin cancers, malignant melanoma was the second most common skin cancer, but at more than double (29.16% vs. 13.1%) comparing to the general Hungarian population. The prominence of skin cancer, particularly melanoma, as the leading type of malignancy underscores the importance of our study. In accordance with previous studies, in the recent study, breast and lung cancers were represented in the second largest number. Interestingly, hematologic malignancies occurred in a much smaller number compared to the literature.

The close temporal relationship of SSc and malignancy was previously described in the literature [16,38]. Considering the duration of SSc, Onishi et al. found that, in the first 12 months after the diagnosis of SSc, the risk of developing a tumor was higher [32]. Launay et al. also reported that most of the malignancies were diagnosed simultaneously or in one year after the diagnosis of SSc [39]. We also observed close onset of tumors to SSc diagnosis; however, a second peak of malignancy frequency was present in our study. The first peak was around the diagnosis of systemic sclerosis and the second peak was 10 years after the diagnosis of SSc. Partouche et al., similarly to our findings, also reported two peaks of tumor onset [16]. In their study, most of the tumors were detected ±5 years from the diagnosis of SSc and 10 years later. The first peak suggests a paraneoplastic feature of SSc and the second peak possibly reflects the importance of risk factors during the disease.

It was shown that men with SSc have a significantly higher risk of tumors, probably due to the larger proportion of smoking [32]. Smoking was more prevalent among male patients; however, male gender was not identified as a risk factor. Smoking individuals appeared to have an increased likelihood of developing malignancies. In the literature, SSc was reported as a risk factor for alcohol-related neoplasms [20,40]. Interestingly, in our study, alcohol consumption occurred primarily in non-cancerous patients. Advanced age at SSc onset was also confirmed to be associated with increased tumor risk [36]. This supports our finding. Cancerous patients were older compared to non-cancerous patients. The protective effect of pregnancy against breast cancer is well-documented [26,41]. However, this effect could not be demonstrated in the present study.

By collecting an extended range of immunoserological positivities, we evaluated the correlation between autoantibodies and malignancies. An increased tumor risk was confirmed in the case of anti-RNAPIII [20,32,34,38,42,43]. Furthermore, some of the studies also reported an association between anti-RNAPIII positivity and close tumor onset to SSc diagnosis [44]. Compared to other autoantibodies, malignancy incidence was higher in anti-RNAPIII positive patients compared to ACA positive patients. In the study by Carbonell et al., ACA positivity was less frequent in cancerous patients, raising the possibility of ACA being protective against tumors [34]. We found that ACA was slightly more common in non-cancerous patients, but this data were not strong enough to draw firm conclusion.

Patients with dcSSc have a major risk of developing tumors, and anti-RNAPIII positivity increases the risk further [35]. Considering the type of neoplasms, Igusa et al. suggested that tumor risk can differ between disease subtypes with the presence of anti-RNAPIII: dcSSc and anti-RNAPIII have higher risk of developing breast cancer and lcSSc with anti-RNAPIII increases the risk of lung cancer. They also claimed that ACA positivity decreases tumor risk. Moinzadeh et al. found that breast cancer was more frequent in anti-RNAPIII positive patients, and it was the only autoantibody that increased tumor risk [45]. In our study, anti-RNAPIII positivity was associated with a 3-fold increase in the risk of malignancy. Neoplasms appeared to be more frequent in patients with dcSSc.

The distribution of anti-PM/Scl positivity among the individual tumor types deserves further consideration. The existing literature has indicated a potential association between anti-PM/Scl autoantibodies and an elevated risk of malignancy [46]. In a recent study, approximately half of these immunoserological positivities were observed in patients with skin cancer, which may indicate a potential association between anti-PM/Scl positivity and cutaneous malignancies. However, due to the low number of cases, no clear conclusion can be drawn, and further investigations are necessary to verify this relationship.

Age at diagnosis of the first tumor and age at the time of SSc diagnosis was the closest in the case of ACA, ATA and anti-RNAPIII positivities. However, a cause-and-effect relationship could not be deduced from this, though it can be clearly stated that these factors are related to each other.

The increased occurrence of hematological malignancies and bladder cancer highlights the potential relevance of exposure to immunosuppressive drugs, particularly cyclophosphamide [27]. In the present study, the analysis of the association between treatment strategies and tumors revealed a relatively low incidence of malignancies among patients receiving azathioprine. In contrast, cyclophosphamide use was associated with a higher, although not statistically significant, malignancy risk, which aligns with the findings reported by Partouche et al. [16].

Patients suffering from SSc have worse survival compared to the general population [47]. Cardiopulmonary complications are the principal causes of mortality, while neoplastic diseases also contribute markedly to the decreased survival. Rate of deaths reached 14% in case of early tumors in our previous publication and 13.1% in the EUSTAR cohort [18,47]. Regarding the Hungarian SSc population, Czirják et al. reported that early malignancy rate was 4.6% and, besides cardiac and renal involvement, dcSSc, advanced age and increased erythrocyte sedimentation rate, the coexistence of SSc and early malignancy worsens survival remarkably (HR 3.23, 95% CI 1.62, 6.32) [18]. In the present study, the rate of early malignancy was observed to be 2.4%. Malignancies accounted for 15.7% of all deaths in patients with SSc. A recent study reported that neoplasms were responsible for 9.7% of all deaths in patients with SSc [48]. Morrisroe et al. reported more than 2-fold increased mortality in SSc patients with tumors compared to patients without tumors [33]. These findings are consistent with our results; however, in our study, the risk of death was four times higher after the diagnosis of malignancy. Nearly half of the patients with neoplasms died due to the tumor or its complications. All these data prove that tumors increase mortality significantly in SSc.

One of the key strengths of this work is that, to the best of our knowledge, this is the first Hungarian study in the literature involving three Hungarian clinical centers that investigates the occurrence of tumors and their influencing factors among Hungarian patients with SSc.

Limitations of this study originate from its retrospective nature, including the lack of data about environmental exposures or pregnancy in the medical records. Potential registry or reporting biases should also be taken into consideration. Additionally, the standardization of alcohol consumption categories was not possible due to retrospective data collection and incomplete patient records.

## 5. Conclusions

Systemic sclerosis patients demonstrate significantly elevated malignancy risk, with neoplasms representing a major contributor to disease-associated mortality. This study successfully identified cutaneous malignancies as the predominant tumor type in the Hungarian SSc cohort, with melanoma emerging as the second most frequent diagnosis. Our findings establish anti-RNAPIII positivity as an independent risk factor for oncogenesis. The marked increase in post-neoplasm mortality underscores the critical need for (1) comprehensive patient education regarding preventive strategies, and (2) implementation of risk-stratified, age-specific surveillance protocols to facilitate early detection. These results provide foundational evidence to guide future development of standardized screening algorithms tailored to the SSc population.

## Figures and Tables

**Table 1:** Exposures in the SSc cohort and their comparison between cancerous and non-cancerous patients and women and men. Regarding smoking, alcohol consumption, exposures, infections and pregnancy, not all data were available for all patients.

	Overall	Tumor		Sex	
Characteristic		With tumor	Without tumor	female	male
	*n* = 541	*n* = 85	*n* = 456	*n* = 463	*n* = 78
Smoking	142/353 (40.2%)	24/57 (42.1%)	118/296 (39.9%)	103/292 (35.3%)	39/61 (63.9%) ***
Alcohol consumption	*n*= 326	*n* = 54	*n* = 272	*n* = 270	*n* = 56
*no*	289 (88.7%)	46 (85.2%)	243 (89.3%)	243 (90.0%)	46 (82.1%)
*sometimes*	1 (0.3%)	1 (1.9%)	0 (0%)	1 (0.4%)	0 (0%)
*occasionally*	4 (1.2%)	4 (7.4%)	0 (0%)	4 (1.5%)	1 (1.8%)
*regularly*	32 (9.8%)	3 (5.6%)	29 (10.7%) ***	23 (8.5%)	9 (16.1%)
Organic solvents	12/156 (7.7%)	1/35 (2.9%)	11/121 (9.1%)	8/132 (6.1%)	4/24 (16.7%)
Silica exp.	0/153 (0%)	0/35 (0%)	0/118 (0%)	0/129 (0%)	0/24 (0%)
Vinyl-cloride exp.	0/153 (0%)	0/35 (0%)	0/118 (0%)	0/129 (0%)	0/24 (0%)
Pregnancy	282/304 (92.8%)	45/49 (91.8%)	237/255 (92.9%)		

Significant *p*-values comparing patients with malignancy versus without malignancy and female versus male are shown as follows: ***: *p* < 0.001. exp.: exposure.

**Table 2:** Univariate and multivariate logistic regression for the investigation of tumor risk factors.

	Variable	Model 1 OR (95% CI)	Model 2 OR (95% CI)	Model 3 OR (95% CI)
**Demographics and SSc characteristics**	Female (reference: male)	1.20 (0.52, 3.27)		
	dcSSc (reference: lcSSc)	1.44 (0.78, 2.63)		
	Age at the diagnosis of SSc	1.01 (0.99, 1.04)		
	Duration of SSc (outcome)	1.00 (0.96, 1.03)		
**Antibody profile**	ANA (+)	2.17 (0.96, 5.84)	1.57 (0.51, 5.93)	1.55 (0.50, 5.88)
	Anti-topoisomerase I (+)	1.36 (0.69, 2.58)	2.34 (0.94, 5.84)	2.44 (0.92, 6.58)
	Anti-RNAPIII (+)	**3.18 (1.09, 8.20)**	**4.33 (1.08, 15.1)**	**4.72 (1.12, 17.6)**
	Anti-centromere (+)	0.68 (0.23, 1.66)	1.25 (0.26, 4.40)	1.22 (0.23, 5.02)
	Anti-Ro52 (+)	0.75 (0.30, 1.65)	1.01 (0.32, 2.76)	1.08 (0.33, 2.99)
**Treatment**	MTX	0.66 (0.31, 1.29)	0.49 (0.16, 1.25)	0.53 (0.18, 1.39)
	CYC	0.92 (0.42, 1.86)	0.97 (0.34, 2.47)	1.09 (0.38, 2.83)
**Exposure**	Smoking	1.05 (0.47, 2.27)	1.26 (0.54, 2.91)	1.27 (0.53, 3.00)

Model 1: Univariate logistic regression: demographics and SSc characteristics, antibody profile, treatment and exposure variables; Model 2: Multivariate logistic regression: antibody profile, treatment and exposure variables; Model 3: Multivariate logistic regression: antibody profile, treatment and exposure variables (controlled for: demographics and SSc characteristics variables); OR (95% CI): Odds Ratio (95% confidence interval), significant results highlighted with bold. SSc: systemic sclerosis, dcSSc: diffuse cutaneous systemic sclerosis, lcSSc: limited cutaneous systemic sclerosis, ANA: antinuclear antibodies, anti-RNAPIII: anti-RNA Polymerase III, MTX: methotrexate, CYC: cyclophosphamide.

## Data Availability

Data are contained within the article and Supplementary Materials.

## Supplementary Materials

The following supporting information can be downloaded at: https://www.mdpi.com/article/10.3390/biomedicines13040993/s1. Table S1: Demographic, clinical, serological, treatment and exposure-related characteristics and mortality data of SSc patients with tumors (96 tumors/85 patients) aggregated and according to different tumor types. Table S2: Patient-level and categorized data on the time interval between immunosuppressive/immunomodulatory treatment and tumor diagnosis. Out of 85 tumor patients, 27 patients received at least one type of therapy before tumor diagnosis.
