# Supplementary material for: Malignancy in Systemic Sclerosis: A Multicenter Retrospective Study
Source: Biomedicines. 2025 Apr 19;13(4):993. doi: 10.3390/biomedicines13040993 (PMC12024861; doi:10.3390/biomedicines13040993)
Supplement: Supplementary file 1 [file biomedicines-13-00993-s001.zip › biomedicines-3565014-supplementary.pdf]

Table S1. Demographic, clinical, serological, treatment and exposure-related characteristics and mortality data of SSc patients with tumors (96 tumors/85 patients) aggregated and according to different tumor types

Regarding smoking, alcohol consumption, exposures, infections and pregnancy not all data was available in all patients.

*: p<0.05

SSc: systemic sclerosis, IQR: interquartile range, ANA: antinuclear antibodies, anti-RNAPIII: anti-RNA Polymerase III, CQ: chloroquine, AZA: azathioprine, MTX: methotrexate, MMF: mycophenolate mofetil, CYC: cyclophosphamide, SSZ: sulfasalazine, LEF: leflunomide, APSCT: autologous peripheral stem-cell transplantation, TCZ: tocilizumab, exp.: exposure

Characteristic	Overall N = 96	Skin cancer N = 24	Breast cancer N = 14	Lung cancer N = 14	Urologic cancer N = 10	Head and neck cancers N = 9	Hematologic malignancies N = 8	Gastrointestinal cancers N = 7	Gynecological cancers N = 7	Other cancers N = 3
Sex, n (%)										
female	83 (86.5%)	24 (100%)	14 (100%)	13 (92.9%)	5 (50%)	6 (66.7%)	6 (75%)	5 (71.4%)	7 (100%)	3 (100%)
male	13 (13.5%)	0 (0%)	0 (0%)	1 (7.1%)	5 (50%)	3 (33.3%)	2 (25%)	2 (28.6%)	0 (0%)	0 (0%)
Age at the diagnosis of tumor (years), median (IQR)	62 (53-69)	69 (57-75)	56 (42-63)	61 (55-67)	63 (59-67)	66 (51-68)	60 (53-70)	63 (58-71)	56 (45-63)	47 (43-71)
Age at the closing of database (years), median (IQR)	68 (61-77)	77 (65-80)	68 (59-73)	68 (58-71)	70 (66-72)	65 (59-69)	67 (63-75)	66 (58-71)	67 (60-71)	48 (45-71)
Type of SSc, n (%)										
limited	54 (56.3%)	18 (75%)*	7 (50%)	7 (50%)	2 (20%)	4 (44.4%)	4 (50%)	3 (42.9%)	7 (100%)*	2 (66.7%)
diffuse	42 (43.8%)	6 (25%)	7 (50%)	7 (50%)	8 (80%)	5 (55.6%)	4 (50%)	4 (57.1%)	0 (0%)	1 (33.3%)
Age at the diagnosis of SSc (years), median (IQR)	54 (44-64)	54 (47-65)	53 (42-66)	51 (41-63)	63 (59-68)	52 (39-58)	54 (51-67)	49 (44-60)	55 (49-70)	38 (30-59)
Duration of SSc (years), median (IQR)	12 (5-19)	18 (9-23)	13 (6-18)	10 (5-16)	5 (1-7)	11 (7-20)	12 (5-17)	17 (7-22)	9 (5-20)	12 (9-14)
Antibody positivity, n (%)										
ANA	83 (86.5%)	22 (91.7%)	12 (85.7%)	14 (100%)	7 (70%)	7 (77.8%)	6 (75%)	5 (71.4%)	7 (100%)	3 (100%)
ant-topoisomerase I	28 (29.2%)	6 (25%)	2 (14.3%)	6 (42.9%)	6 (60%)	2 (22.2%)	2 (25%)	3 (42.9%)	1 (14.3%)	0 (0%)
anti-RNAPIII	11 (11.5%)	3 (12.5%)	2 (14.3%)	2 (14.3%)	1 (10%)	1 (11.1%)	1 (12.5%)	1 (14.3%)	0 (0%)	0 (0%)
anti-centromere	16 (16.7%)	5 (20.8%)	2 (14.3%)	4 (28.6%)	0 (0%)	1 (11.1%)	2 (25%)	0 (0%)	2 (28.6%)	0 (0%)
anti-PM/Scl75 or 100	7 (7.3%)	3 (12.5%)	2 (14.3%)	0 (0%)	0 (0%)	1 (11.1%)	0 (0%)	0 (0%)	0 (0%)	1 (33.3%)
anti-Th/To	3 (3.1%)	2 (8.3%)	0 (0%)	0 (0%)	0 (0%)	0 (0%)	1 (12.5%)	0 (0%)	0 (0%)	0 (0%)
anti-NOR90	1 (1%)	1 (4.2%)	0 (0%)	0 (0%)	0 (0%)	0 (0%)	0 (0%)	0 (0%)	0 (0%)	0 (0%)
anti-fibrillarin	2 (2.1%)	0 (0%)	0 (0%)	0 (0%)	1 (10%)	0 (0%)	0 (0%)	1 (14.3%)	0 (0%)	0 (0%)
anti-Ku	2 (2.1%)	0 (0.0%)	0 (0.0%)	0 (0.0%)	1 (10.0%)	0 (0.0%)	0 (0.0%)	0 (0.0%)	1 (14.3%)	0 (0.0%)
anti-Ro52	18 (18.8%)	5 (20.8%)	3 (21.4%)	2 (14.3%)	1 (10.0%)	2 (22.2%)	2 (25.0%)	1 (14.3%)	2 (28.6%)	0 (0.0%)
negative	4 (4.2%)	0 (0%)	1 (7.1%)	0 (0%)	1 (10%)	0 (0%)	2 (25%)	0 (0%)	0 (0%)	0 (0%)
Treatment before diagnosis of tumor, n (%)										
CQ	3 (3.1%)	0 (0%)	0 (0%)	0 (0%)	0 (0%)	1 (11.1%)	0 (0%)	0 (0%)	2 (28.6%)	0 (0%)
AZA	3 (3.1%)	1 (4.2%)	1 (7.1%)	0 (0%)	0 (0%)	1 (11.1%)	0 (0%)	0 (0%)	0 (0%)	0 (0%)
MTX	18 (18.8%)	6 (25%)	3 (21.4%)	3 (21.4%)	1 (10%)	1 (11.1%)	0 (0%)	1 (14.3%)	1 (14.3%)	2 (66.7%)
MMF	5 (5.2%)	0 (0%)	0 (0%)	4 (28.6%)	0 (0%)	1 (11.1%)	0 (0%)	0 (0%)	0 (0%)	0 (0%)
CYC	13 (13.5%)	2 (8.3%)	3 (21.4%)	4 (28.6%)	2 (20%)	1 (11.1%)	0 (0%)	1 (14.3%)	0 (0%)	0 (0%)
SSZ	3 (3.1%)	1 (4.2%)	0 (0%)	0 (0%)	0 (0%)	1 (11.1%)	0 (0%)	0 (0%)	1 (14.3%)	0 (0%)
LEF	2 (2.1%)	1 (4.2%)	0 (0%)	0 (0%)	0 (0%)	0 (0%)	0 (0%)	1 (14.3%)	0 (0%)	0 (0%)
APSCT	3 (3.1%)	2 (8.3%)	0 (0%)	0 (0%)	0 (0%)	0 (0%)	1 (12.5%)	0 (0%)	0 (0%)	0 (0%)
TCZ	4 (4.2%)	0 (0%)	0 (0%)	2 (14.3%)	1 (10%)	1 (11.1%)	0 (0%)	0 (0%)	0 (0%)	0 (0%)
Exposures, n/N (%)										
Smoking	29/65 (44.6%)	4/10 (40%)	4/8 (50%)	6/13 (46.2%)	4/8 (50%)	3/6 (50%)	1/6 (16.7%)	3/6 (50%)	2/5 (40%)	2/3 (66.7%)
Alcohol consumption	N = 62	N =11	N = 8	N = 11	N = 6	N = 6	N = 6	N = 6	N = 5	N = 3
no	52 (83.9%)	11 (100%)	5 (62.5%)	10 (90.9%)	4 (66.7%)	5 (83.3%)	5 (83.3%)	6 (100%)	4 (80%)	2 (66.7%)
sometimes	1 (1.6%)	0 (0%)	0 (0%)	0 (0%)	0 (0%)	0 (0%)	0 (0%)	0 (0%)	1 (20%)	0 (0%)
occasionally	6 (9.6%)	0 (0%)	2 (25%)	1 (9.1%)	2 (33.3%)	0 (0%)	0 (0%)	0 (0%)	0 (0%)	1 (33.3%)
regularly	3 (4.8%)	0 (0%)	1 (12.5%)	0 (0%)	0 (0%)	1 (16.7%)	1 (16.7%)	0 (0%)	0 (0%)	0 (0%)
Organic solvents	1/39 (2.6%)	0/6 (0%)	0/5 (0%)	1/8 (12.5%)	0/3 (0%)	0/3 (0%)	0/2 (0%)	0/5 (0%)	0/4 (0%)	0/3 (0%)
Silica exp.	0/39 (0%)	0/6 (0%)	0/5 (0%)	0/8 (0%)	0/3 (0%)	0/3 (0%)	0/2 (0%)	0/5 (0%)	0/4 (0%)	0/3 (0%)
Vinyl-chloride exp.	0/39 (0%)	0/6 (0%)	0/5 (0%)	0/8 (0%)	0/3 (0%)	0/3 (0%)	0/2 (0%)	0/5 (0%)	0/4 (0%)	0/3 (0%)
Pregnancy	47/51 (92.2%)	7/8 (87.5%)	9/9 (100%)	9/10 (90%)	3/3 (100%)	3/3 (100%)	4/4 (100%)	4/4 (100%)	5/7 (71.4%)	3/3 (100%)
Mortality, n (%)										
overall deaths	25 (26%)	8 (33.3%)	3 (21.4%)	5 (35.7%)	2 (20%)	2 (22.2%)	1 (12.5%)	4 (57.1%)	0 (0%)	0 (0%)
in tumor positive patients	11 (11.5%)	0 (0%)	1 (7.1%)	5 (35.7%)	1 (10%)	1 (11.1%)	0 (0%)	3 (42.9%)	0 (0%)	0 (0%)

Table S2. Patient-level and categorized data on the time interval between immunosuppressive/immunomodulatory treatment and tumor diagnosis. Out of 85 tumor patients, 27 patients received at least one type of therapy before tumor diagnosis

CQ: chloroquine, HCQ: hydroxychloroquine, AZA: azathioprine, MTX: methotrexate, MMF: my-cophenolate mofetil, CYC: cyclophosphamide, SSZ: sulfasalazine, LEF: leflunomide, APSCT: autologous peripheral stem-cell trans-plantation, TCZ: tocilizumab

Patients	CQ/HCQ	AZA	MTX	MMF	CYC	SSZ	LEF	APSCT	TCZ
Patient 01	-	-	-	-	-	-	-	4 years	-
Patient 02	-	7 years	6 years	-	-	-	-	-	-
Patient 03	2 years	-	1 year	-	-	-	-	-	-
Patient 04	-	-	-	-	13 years	-	-	-	-
Patient 05	-	-	-	-	3 years	-	-	-	-
Patient 06	7 years	6 years	-	6 years	7 years	-	-	-	-
Patient 07	-	14 years	4 years	-	13 years	-	-	-	-
Patient 08	-	-	5 years	-	-	-	-	-	-
Patient 09	-	-	-	-	1 year	-	-	-	-
Patient 10	-	-	4 years	-	-	-	-	-	-
Patient 11	-	-	9 years	-	10 years	-	-	-	-
Patient 12	-	-	-	3 years	13 years	-	-	-	-
Patient 13	-	-	4 years	-	5 years	-	-	-	3 years
Patient 14	-	-	9 years	-	10 years	-	-	-	-
Patient 15	-	-	2 years	-	-	-	-	-	-
Patient 16	-	-	13 years	-	-	-	13 years	-	-
Patient 17	-	-	4 years	3 years	4 years	-	-	-	2 years
Patient 18	10 years	-	-	-	-	10 years	-	-	-
Patient 19	-	-	1 year	-	10 years	-	-	-	-
Patient 20	-	-	-	-	-	-	-	3 years	-
Patient 21	-	-	8 years	-	-	-	-	-	-
Patient 22	-	-	19 years	-	-	-	-	-	-
Patient 23	-	-	-	4 years	-	-	-	-	-
Patient 24	-	-	10 years	7 years	10 years	-	-	-	3 years
Patient 25	-	-	-	-	-	20 years	-	-	-
Patient 26	-	-	-	-	20 years	-	-	-	-
Patient 27	-	-	-	-	-	-	-	-	5 years
**Categories**	***n* = 3**	***n* = 3**	***n* = 15**	***n* = 5**	***n* = 13**	***n* = 2**	***n* = 1**	***n* = 2**	***n* = 4**
≤5 years	1	0	8	3	4	0	0	2	4
6-10 years	2	2	5	2	5	1	0	0	0
>10 years	0	1	2	0	4	1	1	0	0
